# The microRNA cluster miR-106b~25 regulates adult neural stem/progenitor cell proliferation and neuronal differentiation

**DOI:** 10.18632/aging.100285

**Published:** 2011-02-20

**Authors:** Jamie O. Brett, Valérie M. Renault, Victoria A. Rafalski, Ashley E. Webb, Anne Brunet

**Affiliations:** ^1^ Department of Genetics, Stanford University School of Medicine; Stanford, CA 94305, USA; ^2^ Neurosciences Program, Stanford University School of Medicine, Stanford, CA 94305, USA

**Keywords:** aging, neural stem cells, microRNAs, FoxO transcription factors, insulin signaling, neuronal differentiation

## Abstract

In adult mammals, neural stem cells (NSCs) generate new neurons that are important for specific types of learning and memory. Controlling adult NSC number and function is fundamental for preserving the stem cell pool and ensuring proper levels of neurogenesis throughout life. Here we study the importance of the microRNA gene cluster miR-106b~25 (miR-106b, miR-93, and miR-25) in primary cultures of neural stem/progenitor cells (NSPCs) isolated from adult mice. We find that knocking down miR-25 decreases NSPC proliferation, whereas ectopically expressing miR-25 promotes NSPC proliferation. Expressing the entire miR-106b~25 cluster in NSPCs also increases their ability to generate new neurons. Interestingly, miR-25 has a number of potential target mRNAs involved in insulin/insulin-like growth factor-1 (IGF) signaling, a pathway implicated in aging. Furthermore, the regulatory region of miR-106b~25 is bound by FoxO3, a member of the FoxO family of transcription factors that maintains adult stem cells and extends lifespan downstream of insulin/IGF signaling. These results suggest that miR-106b~25 regulates NSPC function and is part of a network involving the insulin/IGF-FoxO pathway, which may have important implications for the homeostasis of the NSC pool during aging.

## INTRODUCTION

New neurons are generated in the mammalian brain throughout adult life. Slowly dividing and self-renewing neural stem cells (NSCs) are present in the subventricular zone (SVZ) of the lateral ventricles and in the subgranular zone (SGZ) of the hippocampal dentate gyrus. NSCs generate rapidly proliferating neural progenitor cells that ultimately differentiate to produce thousands of new neurons each day in adult rats [1]. The progeny of SVZ NSCs migrate to the olfactory bulb where they mature into inhibitory interneurons with roles in olfactory learning and memory [2]. SGZ NSCs produce excitatory neurons that integrate into the dentate gyrus and are critical for certain types of hippocampus-dependent learning and memory [1,3]. Neurogenesis declines with age [4-6] and is impaired by various types of stress [7] and brain inflammation [8]. Exercise and environmental enrichment increase neurogenesis, and can reverse the effects of aging [9,10] and stress [11]. Excessive NSC proliferation, however, can promote functional exhaustion of these cells [12-14] and in some cases can lead to glioma, a form of brain cancer [15-17]. Thus, regulation of NSC proliferation and differentiation is pivotal for adult brain homeostasis and is disrupted during aging.

Intrinsic and extrinsic factors regulate NSC function largely by directing changes in gene expression. A number of transcription factors and chromatin modifiers control gene expression in adult NSCs, thereby affecting NSC number and ability to differentiate into multiple cell types. These regulators include the polycomb member Bmi1 [18-20], the transcriptional repressor Tlx [21,22], and the FoxO family of transcription factors [13,14]. MicroRNAs (miRNAs) represent an additional layer of gene expression control and have recently emerged as key regulators of embryonic and adult stem cells [23,24]. miRNAs are single-stranded ~23-nucleotide RNA molecules that are usually derived from long primary host transcripts [25]. In the cytoplasm, miRNAs direct destabilization and translational repression of target mRNAs by binding sites usually in mRNA 3' untranslated regions. This miRNA-directed downregulation of gene expression generally requires a complementary match between the mRNA target site and the second to seventh nucleotides of the miRNA 5' end (the “seed sequence”). It also depends on other regions of complementary pairing between the mRNA site and the miRNA, the presence of other miRNA-targeted sites, and the mRNA structure at this region [26]. Several hundred miRNAs have been identified in humans and mice [27]. As each miRNA potentially targets hundreds of different mRNAs [28], miRNAs can coordinate cell behaviors by fine-tuning gene expression [26,29].

A number of miRNAs recently have been found to regulate adult NSCs in vivo and in culture [30]. For example, two miRNAs, let-7b and miR-9, inhibit NSC proliferation and promote neuronal differentiation by suppressing Tlx and the oncogenic chromatin regulator Hmga2 [31-33]. In addition, miR-124 promotes differentiation of SVZ NSCs into neuroblasts by repressing the expression of the transcription factor Sox9 [34]. Finally, miR-184 and miR-137 trigger NSC proliferation and inhibit differentiation by repressing the NSC fate-regulator Numblike [35] and the polycomb methyltransferase Ezh2 [36], respectively. Thus, miR-124, miR-9, and let-7b elicit NSC differentiation, while miR-184 and miR-137 increase proliferation at the expense of differentiation potential. miRNAs that promote the expansion of NSCs while maintaining their ability to differentiate have not yet been identified.

The miRNAs in the miR-17 family are attractive candidates for this function. Specific miR-17 family members are overexpressed in a variety of cancers, including glioma and glioblastoma brain cancers [37-41], and promote cancer cell proliferation and survival [42-45]. Furthermore, in embryonic stem cells, miR-17 family members are repressed by the REST neuronal gene silencer [46], which negatively regulates neurogenesis [47]. miR-17 member expression in the brain declines between late embryonic and postnatal life [48], which correlates with the decline in neurogenesis that occurs during this period [49,50]. These results suggest that miR-17 members may be involved in promoting both proliferation and neurogenesis.

The miR-17 family consists of three paralogous polycistronic clusters on different chromosomes: miR-17~92 (miR-17, miR-18a, miR-19a, miR-20a, miR-19b-1, and miR-92a-1), miR-106b~25 (miR-106b, miR-93, and miR-25), and miR-106a~363 (miR-106a, miR-18b, miR-20b, miR-19b-2, miR-92a-2, and miR-363). Members of each cluster belong to one of four groups with similar seed sequences and therefore similar mRNA targets [51]. Within the miR-17 family, members of the miR-106b~25 cluster (miR-106b, miR-93, and miR-25) appear to be the most strongly expressed in the adult brain [27,52]. Further suggesting a link between miR-106b~25 and neurogenesis, expression of the host gene for miR-106b~25, Mcm7, is reduced in a mouse model of Down syndrome with diminished numbers of neural progenitor cells and neurogenesis [53].

Interestingly, the miR-106b~25 genomic locus contains a consensus binding sequence for the FoxO transcription factors. FoxO factors are inhibited by the insulin/insulin-like growth factor-1 (IGF) signaling pathway [54-56] and have emerged as regulators of adult NSCs both in vitro and in vivo [13,14]. The FoxO family promotes longevity in a range of species [57-59] and is involved in nematode lifespan regulation by the miRNA lin-4 [60]. FoxO3, one member of the FoxO family, has recently been associated with extreme longevity in humans [61-65]. The presence of a FoxO binding sequence in the miR-106b~25 genomic locus raises the possibility of an interaction between this miRNA cluster and the insulin/IGF-FoxO pathway in mammals.

Here we use primary cultures of neural stem/progenitor cells (NSPCs) from adult mice to show that miR-106b~25 promotes NSPC proliferation. Knocking down miR-25 decreases NSPC proliferation, and ectopically expressing miR-25 or the entire miR-106b~25 cluster increases proliferation. In NSPCs induced to differentiate, overexpressing miR-106b~25 enhances differentiation toward the neuronal lineage. We find that potential miR-25 target mRNAs are overrepresented in insulin/IGF signaling. Furthermore, we show that FoxO3 occupies a binding site near the promoter for miR-106b~25 in NSPCs, raising the possibility of a FoxO-miR-106b~25 feedback loop. Together, these results suggest that miR-106b~25 modulates adult NSPC proliferation and neuronal differentiation, which may have crucial implications for the maintenance of adult neurogenesis.

## RESULTS

### miR-106b, miR-93, and miR-25 are expressed in adult NSPC cultures

**Figure 1.**
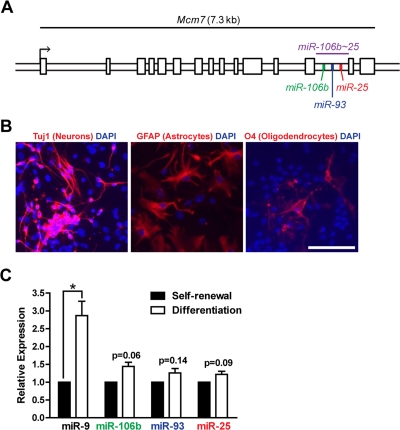
The miR-106b_~_25 cluster is expressed in adult NSPCs in culture. (**A**) Genomic locus of the mouse miR-106b~25 cluster and its host gene, Mcm7. (**B**) NSPCs (age 12 weeks, passage 2) were grown in multi-lineage differentiation conditions (no EGF or bFGF, with 1% FBS) for 7 days and then stained for Tuj1 (a marker of neurons), GFAP (a marker of astrocytes), or O4 (a marker of oligodendrocytes). Scale bar: 100 μm. (**C**) miRNA expression was determined by RT-qPCR in NSPCs in self-renewal conditions (with EGF and bFGF, no FBS) or differentiation conditions (no EGF or bFGF, with 1% FBS) for 4 days. Mean and SEM of gene expression relative to self-renewal conditions for 3 independent NSPC cultures (age 12 weeks, passage 2) are shown. One-sample two-tailed t-test, *: p<0.05.

We examined the expression levels of the miR-106b~25 cluster members (miR-106b, miR-93, and miR-25; Figure 1A) in self-renewing or differentiating NSPCs isolated from young adult (3 month-old) mice. After the first passage in culture, NSPCs were placed in self-renewal conditions or in differentiation conditions known to give rise to astrocytes, neurons, and oligodendrocytes [14,66]. We confirmed differentiation of NSPCs into these cell types by staining for markers of astrocytes (GFAP-positive), neurons (Tuj1-positive), and oligodendrocytes (O4-positive) [67] after seven days of differentiation (Figure 1B). We then tested the expression of miR-106b~25 by RT-qPCR in self-renewing and differentiating NSPCs (Figure 1C). We found that miR-106b, miR-93, and miR-25 were all expressed in self-renewing NSPCs. Expression of these miRNAs was not significantly changed by multi-lineage differentiation, although these miRNAs tended to be slightly upregulated during differentiation. In contrast, miR-9, a miRNA known to be induced by NSPC differentiation [33], was significantly upregulated in differentiating NSPCs. Together, these results indicate that miR-106b~25 is expressed in both self-renewing and differentiating adult NSPCs.

### miR-25 is important for adult NSPC proliferation

**Figure 2.**
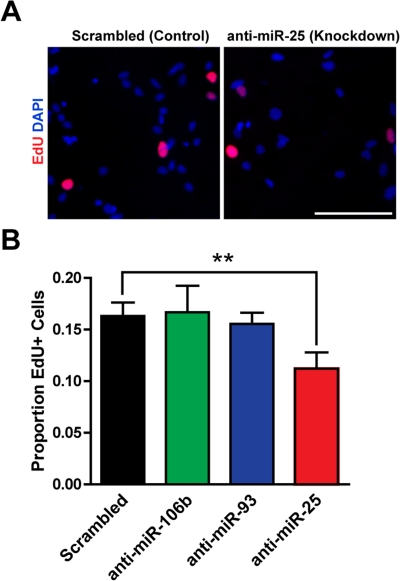
miR-25 is necessary for adult NSPC prolifer-ation. NSPCs were transfected to knock down miR-106b, miR-93, or miR-25 or were transfected with a scrambled control oligonucleotide. Two days after transfection, NSPCs were incubated with EdU for 1 hour and then immediately fixed for analysis. (**A**) Representative photos for control knockdown and miR-25 knockdown. Scale bar: 100 μm. (**B**) Mean and SEM of the proportion of EdU+ cells for each condition, for experiments on 5 independent NSPC cultures (age 8-14 weeks, passage 3-7). Paired two-tailed t-test, **: p<0.01.

We next tested whether miR-106b~25 is important for adult NSPC proliferation in self-renewal conditions. To inhibit miR-106b~25, we transfected NSPCs with locked nucleic acid (LNA)-modified oligonucleotides antisense to miR-106b, miR-93, or miR-25, or with a scrambled control LNA oligonucleotide. We assessed incorporation of the thymidine analog 5-ethynyl-deoxyuridine (EdU) in NSPCs transfected with LNA probes antisense to each of the miRNAs in the miR-106b~25 cluster or with control LNA probes. We found that miR-25 knockdown decreased EdU incorporation in NSPCs by 45% (p=0.005), whereas miR-106b or miR-93 knockdown did not significantly affect EdU incorporation in NSPCs (Figure 2). These results indicate that within the miR-106b~25 cluster, miR-25 is the most important for NSPC proliferation.

### Ectopic expression of miR-25 promotes proliferation in adult NSPCs

**Figure 3.**
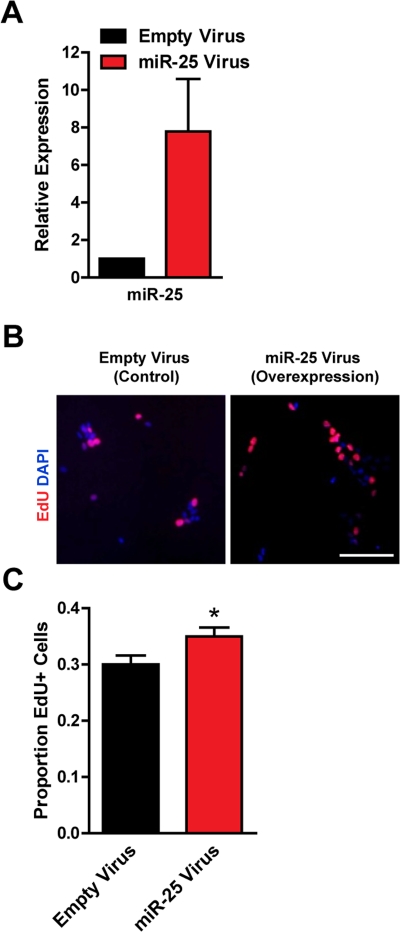
Expression of miR-25 enhances adult NSPC proliferation. NSPCs were infected with an empty control retrovirus (expressing a GFP marker only) or a retrovirus expressing miR-25. NSPCs were grown to full neurospheres for about 1 week after infection before miRNA expression and proliferation were analyzed. (**A**) miR-25 expression was assessed with RT-qPCR in control versus miR-25-overexpressing NSPCs. Mean and SEM of 2 independent NSPC cultures (age 12 weeks, passage 2-5) are shown. (**B**) Representative photos for each condition. Scale bar: 100 μm. (**C**) Control and miR-25-overexpressing NSPCs were dissociated and incubated with EdU for 1 hour. Mean and SEM of the proportion of EdU+ cells for each condition, for experiments on 4 independent NSPC cultures (age 12 weeks, passage 3-6), are shown. Paired two-tailed t-test, *: p<0.05.

To test if miR-25 could promote proliferation in adult NSPCs, we ectopically expressed miR-25 in NSPCs using a retroviral vector containing the miR-25 precursor and green fluorescent protein (GFP). We verified by RT-qPCR that miR-25 was overexpressed, on average by 8-fold, in NSPCs after miR-25 retrovirus infection (Figure 3A). We found that ectopic miR-25 expression increased NSPC incorporation of EdU by 18% compared to the GFP-only control (p=0.04; Figure 3B,C).

**Figure 4.**
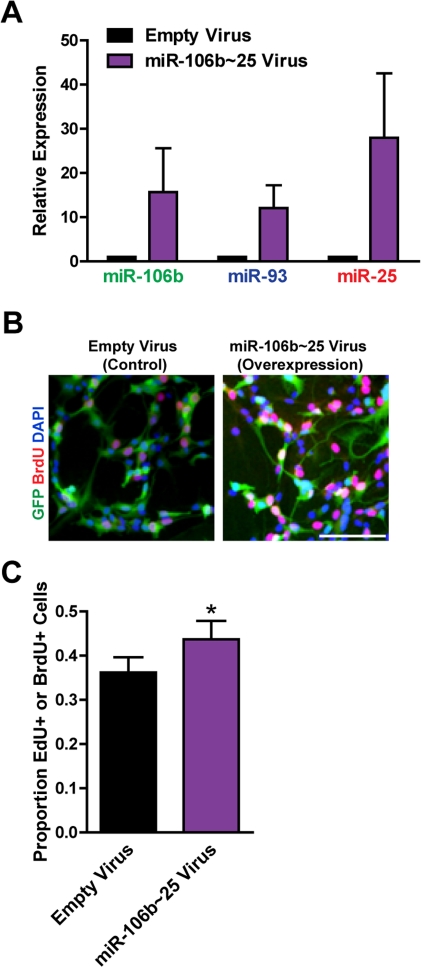
Expression of the entire miR-106b~25 cluster also enhances adult NSPC proliferation. NSPCs were infected with an empty control retrovirus (expressing a GFP marker only) or a retrovirus expressing miR-106b, miR-93, and miR-25 simultaneously (miR-106b~25). NSPCs were grown to full neurospheres for about 1 week after infection before miRNA expression and proliferation were analyzed. (**A**) miR-106b, miR-93, and miR-25 expression was assessed with RT-qPCR in control versus miR-106b~25-overexpressing NSPCs. Mean and SEM of 4 independent NSPC cultures (age 12-14 weeks, passage 5-14) are shown. (**B**) Representative photos for each condition. Scale bar: 100 μm. (**C**) Control and miR-106b~25-overexpressing NSPCs were dissociated and incubated with EdU or BrdU for 1 hour. Mean and SEM of the proportion of EdU+ or BrdU+ cells for each condition, for 6 experiments on independent NSPC cultures (age 12-14 weeks, passage 3-14), are shown. Paired two-tailed t-test, *: p<0.05.

### Expression of the miR-106b~25 cluster promotes neuronal differentiation of adult NSPCs

**Figure 5.**
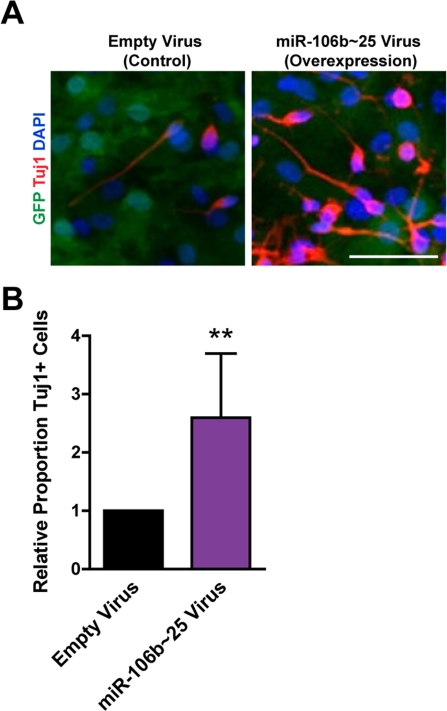
miR-106b_~_25 enhances neurogenesis in culture. NSPCs were infected with an empty control virus or virus to overexpress miR-106b_~_25. Three days after infection, NSPCs were placed in differentiation conditions for 7 days, and then stained for Tuj1, a marker of neurons. (**A**) Representative photos for each condition. Scale bar: 50 μm. (**B**) Mean and SEM of the proportion of Tuj1+ cells (total Tuj1+ cells/total DAPI-stained nuclei) normalized to control infection, for experiments on 4 independent NSPC cultures (age 12 weeks, passage 2), are shown. Paired two-tailed t-test, **: p<0.01.

We examined how miR-106b~25 influences the generation of neurons from NSPCs during multi-lineage differentiation in culture. Because the short-term nature of LNA-mediated miRNA knockdown is not compatible with the duration of NSPC differentiation, we examined the effect of retrovirus overexpression of miR-106b~25 on neuronal differentiation. We infected NSPCs with retroviruses expressing miR-106b~25 or control retroviruses and then differentiated these cells for seven days. We stained cells for Tuj1, a marker of neurons, and determined the proportion of Tuj1-positive cells (Figure 5). Although infected NSPCs formed relatively few neurons - probably a consequence of the toxicity of the infection - we found that compared to control infection, miR-106b~25 expression consistently increased the proportion of Tuj1-positive cells, on average from 0.3% to 0.9% (2.6-fold; p=0.005). These results indicate that ectopic expression of miR-106b~25 can enhance neurogenesis in culture.

### miR-25 has a number of predicted targets in the TGFβ and insulin/IGF-FoxO pathways

**Figure 6.**
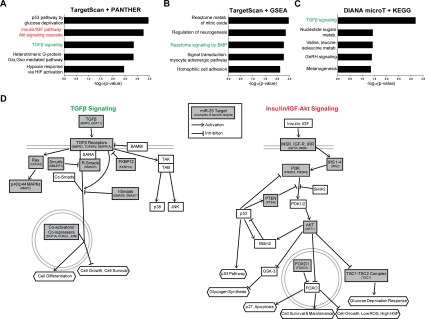
miR-25 targets genes involved in TGFβ and insulin/IGF signaling. (**A**) The PANTHER gene classification program was used to analyze TargetScan-predicted conserved targets for mouse miR-25 (~600 targets total). Shown are the top 5 biological pathways (ordered by Bonferroni-corrected binomial test p-values). (**B**) The GSEA program was used to analyze the same TargetScan-predicted target list as in (A), using the Canonical Pathways and GO Gene Sets categories. Shown are the top 5 categories (ordered by hypergeometric distribution-generated p-values). (**C**) The DIANA-microT program was used to generate a stringent list of mouse miR-25 targets. Shown are the top KEGG categories (ordered by Pearson's chi-square test p-values). (**D**) Pathway diagrams based on those in PANTHER Pathways for TGFβ and insulin/IGF-Akt signaling pathways, modified for simplicity and with select miR-25 predicted targets listed.

**Figure 7.**
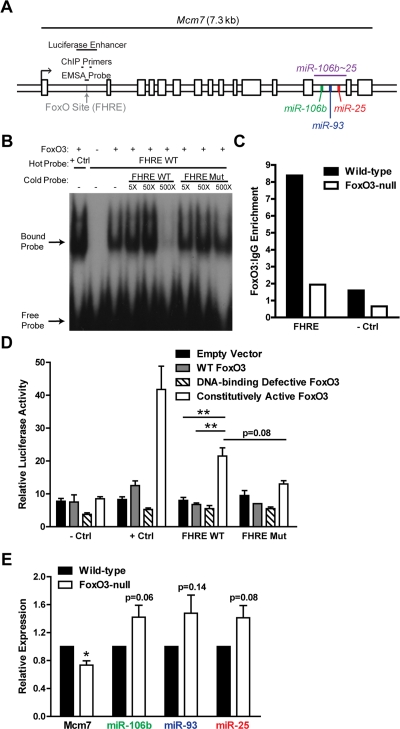
FoxO3 binds to a site in the first intron of miR-106b~25/Mcm7. (**A**) Location of the FoxO binding site (FHRE) within the first intron of the miR-106b~25/Mcm7 gene, and the sequence locations used for EMSA, ChIP, and luciferase experiments. (**B**) EMSA with recombinant FoxO3-GST and a radioactively-labeled (hot) probe corresponding to the FoxO binding site in miR-106b~25/Mcm7 (FHRE WT). + Ctrl: FoxO3-GST incubated with a probe for a known FoxO binding site. The specificity of the interaction was tested by increasing amounts of unlabeled (cold) probe or cold probe with mutations in the FoxO consensus binding sequence (FHRE Mut). (**C**) Wild-type and FoxO3-null NSPCs were dissociated and the next day treated with 4 hours growth factor removal followed by addition of LY294002 for 1 hour. Antibodies to FoxO3 or control IgG antibodies were used for ChIP. qPCR was used to assess the enrichment of FHRE and of a negative control site (- Ctrl). Shown is the relative enrichment for 1 experiment (age 12 weeks, passage 10). These results were confirmed in ChIP-Seq studies (Webb et al. submitted). (**D**) HEK 293T cells were co-transfected with a plasmid to express FoxO3 (empty control, wild-type FoxO3, FoxO3 lacking the DNA binding domain, or constitutively nuclear FoxO3), a firefly luciferase reporter containing FHRE with or without the FoxO consensus sequence mutated, and a Renilla luciferase reporter to normalize for transfection efficiency. As a positive control, a luciferase reporter containing a known FoxO3-activated site was used (+ Ctrl); as a negative control, a luciferase reporter without an enhancer site was used (- Ctrl). Luciferase activity was assessed two days after transfection. Mean and SEM for 4 independent experiments (- Ctrl, + Ctrl, and FHRE WT) or 2 independent experiments (FHRE Mut) are shown. Unpaired two-tailed t-test, **: p<0.01. (**E**) NSPCs from wild-type and FoxO3-null mice were isolated and cultured. Total RNA was collected, and the levels of mature miR-106b~25 members (relative to 5S RNA) and Mcm7 mRNA (relative to β-actin mRNA) were assessed by RT-qPCR. Mean and SEM of the FoxO3-null/wild-type fold change for 5-6 independent cultures (age 10-13 weeks, passage 2-5) are shown. One-sample two-tailed t-test, *: p<0.05.

We next sought to identify the molecular networks involving miR-25, the main miR-106b~25 member controlling NSPC proliferation. Computational algorithms have been developed to predict miRNA binding sites on target mRNA transcripts, based on miRNA-target site complementarity, site context, and site conservation [26]. To examine miR-25 targets through multiple bioinformatics approaches, we first used the TargetScan program [68] to predict the conserved mRNA targets of miR-25 (~600 targets) and then used the gene classification programs PANTHER [69,70] (Figure 6A) or GSEA [71] (Figure 6B) to associate biological processes and gene sets with these targets. In a parallel approach, we used the DIANA-miRPath program [72] to predict miR-25 targets (~150) with the DIANA-microT-3.0-Strict algorithm [73] followed by comparison with the Kyoto Encyclopedia of Genes and Genomes (KEGG) biological pathways [74] (Figure 6C). A number of interesting molecular networks were enriched for miR-25 targets, including p53 signaling, hypoxia signaling, and nitric oxide signaling, which are all important for NSC maintenance and activity [75-77]. Two signaling pathways in particular stood out from this target analysis: transforming growth factor β (TGFβ)/bone morphogenic protein (BMP) signaling, which was enriched for miR-25 targets in all three bioinformatics approaches, and insulin/IGF signaling, which was enriched for miR-25 targets in the TargetScan-PANTHER analysis (Figure 6D). TGFβ signaling has been shown to inhibit adult NSC proliferation and neurogenesis [78,79], suggesting that miR-25 might promote NSC proliferation and neuronal differentiation by repressing TGFβ signaling. Activation of the insulin/IGF pathway, which inhibits FoxO factors [55], increases NSPC proliferation and self-renewal [80-83], and FoxO factors are necessary to maintain the relatively quiescent pool of adult NSCs [13,14]. The observation that the insulin/IGF-FoxO pathway is enriched for miR-25 targets is especially pertinent because the genomic locus of miR-106b~25 contains a conserved FoxO binding sequence (Figure 7A). Furthermore, there is crosstalk between TGFβ signaling and the insulin/IGF-FoxO pathway in nematode longevity, mammalian stem cells, and cancer cells [84-86]. Taken together, these results suggest that modulation of the TGFβ and insulin/IGF signaling pathways may mediate part of the effects of miR-25 in NSPCs.

### The transcription factor FoxO3 binds to a site in the first intron of miR-106b~25/Mcm7

The precursors of miR-106b~25 members are all located in the thirteenth intron of the protein-coding gene Mcm7, a member of a DNA helicase family required for DNA replication [87]. The first intron of the Mcm7 gene contains a conserved core binding sequence (TTGTTTAC) for the FoxO proteins [88,89] (Figure 7A). As the FoxO factors, particularly FoxO3, are important for NSC self-renewal, proliferation, and differentiation [13,14], we tested whether FoxO3 could bind to this site in the first intron of miR-106b~25/Mcm7. We performed an electrophoretic mobility shift assay (EMSA) in which recombinant FoxO3 was incubated with a 38-bp probe containing the FoxO binding sequence in the miR-106b~25 genomic locus. We found that FoxO3 caused a band shift of this probe, showing that FoxO3 directly binds this site in vitro (Figure 7B). To determine if FoxO3 is present at the binding site at the miR-106b~25 locus in NSPCs in the context of endogenous chromatin, we performed FoxO3 chromatin immunoprecipitation (ChIP) on NSPCs treated with brief growth factor removal and the PI3K inhibitor LY294002, to activate endogenous FoxO3 (Figure 7C). ChIP-qPCR showed that endogenous FoxO3 occupies the binding site in the first intron of miR-106b~25/Mcm7 in cultured adult NSPCs. This enrichment was not present in FoxO3-null NSPCs, verifying the specificity of the FoxO3 ChIP. These results indicate that FoxO3 is bound at the genomic locus of the miR-106b~25 cluster.

To test if FoxO3 could upregulate the transcription of miR-106b~25/Mcm7, we generated a luciferase reporter construct containing a minimal SV40 promoter and the 500 bp surrounding the FoxO binding site in the first intron of miR-106b~25/Mcm7 (Figure 7A). We co-transfected HEK 293T cells with this reporter construct and with plasmids to express wild-type FoxO3, a DNA-binding-defective inactive form of FoxO3, or constitutively active FoxO3. These luciferase assays revealed that constitutively active FoxO3 enhanced luciferase expression (p=0.002), and this was partly abrogated by mutating the FoxO binding site (p=0.08), indicating that FoxO3 acts as a transcriptional activator at this genomic locus in HEK 293T cells (Figure 7D).

We next investigated whether FoxO3 affects endogenous miR-106b~25 and Mcm7 expression in NSPCs by comparing the expression of miR-106b~25 and Mcm7 in cultured NSPCs from wild-type versus FoxO3-null adult mice (Figure 7E). FoxO3-null NSPCs had decreased abundance of Mcm7 mRNA (p=0.01), indicating that Mcm7 is a target gene of FoxO3. However, FoxO3-null NSPCs did not display decreased expression of the mature forms of miR-106b, miR-93, and miR-25, suggesting that FoxO3 does not directly upregulate miR-106b~25 and might even indirectly repress the expression of this cluster. Together, these results suggest a complex regulation in which FoxO3 activates the transcription of miR-106b~25/Mcm7, but may repress miR-106b~25 abundance, perhaps by a posttranscriptional mechanism or by acting at a separate promoter for miR-106b~25.

## DISCUSSION

miR-106b~25 members are known to promote cancer cell proliferation and survival [42,44], modulate embryonic stem cell differentiation [90], and promote reprogramming of mouse embryonic fibroblasts into induced pluripotent stem cells [91] - but the importance of miR-106b~25 has not been investigated in an adult stem cell population before. This study examined the role of miR-106b~25 in adult NSPCs. We found that miR-25 knockdown decreases NSPC proliferation, miR-25 or miR-106b~25 overexpression increases adult NSPC proliferation, and miR-106b~25 overexpression promotes neuronal differentiation. Furthermore, FoxO3 binds near the promoter for the host gene of miR-106b~25 and thus has the potential to influence miR-106b~25 expression. These results add to our understanding of the factors regulating NSPC activity and suggest that oncogenic miRNAs could have physiological functions in adult stem cells.

### miR-106b~25 and NSPC proliferation

The effects of miR-106b~25 on adult NSPC proliferation are modest: miR-106b~25 or miR-25 overexpression increased NSPC proliferation by about 1.2-fold, miR-25 knockdown reduced proliferation by about 1.4-fold, and individual miR-106b and miR-93 knockdowns did not affect NSPC proliferation. While these proliferation changes are somewhat smaller than those seen by miR-106b~25 manipulation in carcinoma cells (up to 1.8-fold in similar assays) [42,44], a modest effect of miR-106b~25 on NSPC proliferation could still be important physiologically. While an enforced large increase in NSC proliferation rate could result in tumor initiation [15,17], a weaker increase in proliferation rate could lead to premature stem cell exhaustion [12,13]. On the other hand, fewer divisions could reduce NSC number and neurogenesis [32,92]. Therefore, it is possible that miR-106b~25 overexpression or underexpression, both of which alter NSPC proliferation in culture, could affect long-term NSC function in vivo.

Redundancy within the miR-17 family could dampen the influence of miR-106b~25 on NSPC proliferation in vitro. Knockdown of miR-106b or miR-93, which shares the same mRNA-targeting seed sequence, did not affect proliferation, while knockdown of miR-25, which has a different seed sequence, reduced proliferation. miR-106b and miR-93 might be able to compensate for each other in NSPCs, which could be tested by inhibiting both miRNAs simultaneously. Furthermore, it is possible that NSPCs buffer miR-106b~25 alteration by expressing miR-17 family members from the other paralogous clusters, thereby lessening the relative importance of one or even three miRNAs within this family, or allowing NSPCs to react to changes in miR-106b~25 expression with compensatory changes in miR-17~92 or miR-106a~363 expression. Our findings suggest the idea that compared to cancer cells, stem cells may be more resilient against oncogene perturbation, and therefore more tolerant of certain gene-specific anti-cancer therapies. This may be particularly true for miRNAs, which have been duplicated during animal evolution and tend to have overlapping targets and functions. Such redundancy may have evolved not only so that duplicated miRNAs can be controlled by distinct cis regulatory elements, but perhaps also so that stem cells can absorb fluctuations in gene expression.

### miR-106b~25 in neuronal differentiation

We found that miR-106b~25 promotes both NSPC proliferation in self-renewal conditions and neuron production in differentiation conditions, whereas other miRNAs previously studied in adult NSCs seem to promote one function while inhibiting the other. The mechanism of this effect is still unknown: miR-106b~25 could affect NSPC tendency to produce neurons instead of glia, neuronal progenitor proliferation and survival, and neuron survival. Thus, it remains to be determined whether miR-106b~25 influences neurogenesis by directing cell fate or by regulating cell division and survival in specific cell types.

Adult NSCs decline in number and proliferation, neurogenesis, and self-renewal abilities during aging [5]. Activities that restore NSC activity, such as exercise or environmental enrichment, also restore cognitive performance in aged mice [93,94]. As NSC decline may contribute to cognitive aging, investigating how miR-106b~25 affects neurogenesis will improve our understanding of the molecular mechanisms involved in cognitive aging. While miR-106b~25 knockout mice have no apparent phenotype [52], neurogenesis and learning have not been examined in these mice. It would be worthwhile to investigate how NSCs lacking or overexpressing miR-106b~25 in vivo preserve their numbers and sustain neurogenesis throughout life.

### Potential signaling pathways regulated by miR-25

Deciphering how stem cells sense and respond to tissue integrity and nutrient supply is key to understanding how stem cells maintain tissue homeostasis and how this function changes with age [83,95,96]. Analyzing candidate targets of miR-25 revealed that miR-25 might modulate TGFβ or insulin/IGF signaling at multiple points in each pathway. As TGFβ signaling negatively regulates adult NSC proliferation and neurogenesis [78,79], one way miR-106b~25 might promote these behaviors is by repressing TGFβ signaling in NSPCs. TGFβ Receptor-2 is directly repressed by miR-106b in neuroblastoma cells [97] and by miR-106b and miR-93 in mouse embryonic fibroblasts [91]; thus, one enticing possibility is that TGFβ Receptor-2 is targeted by all miR-106b~25 members in NSPCs. While inhibitory Smads (Smad6 and Smad7) are also predicted miR-25 targets, Smad7-deficient mice have increased adult NSPC proliferation and numbers, which may be due to TGFβ-independent mechanisms [98]. The net functional effect of miR-25 regulation of TGFβ signaling in NSPCs will depend on the relative expression, degree of miR-25 repression, and network connections of each member of the TGFβ pathway in NSPCs.

Activation of the insulin/IGF pathway is sufficient to increase NSPC proliferation and self-renewal [80-83], while FoxO factors are necessary to prevent overproliferation, abnormal differentiation, and long-term depletion of NSCs [13,14]. Thus, another way miR-25 might increase NSPC proliferation is by de-repressing insulin/IGF signaling. Given that PTEN can be a major inhibitor of insulin/IGF signaling [99,100] and is a known target of miR-25 in prostate cancer cells [101], miR-25 may target PTEN to increase insulin/IGF signaling and repress FoxO activity. We cannot exclude the possibility, however, that miR-25 negatively regulates insulin/IGF signaling under some circum-stances, such as by repressing Akt or PI3K.

There may even be crosstalk between the different pathways targeted by miR-25. In nematodes the TGFβ pathway has been shown to genetically interact with the insulin/IGF-FoxO pathway to regulate lifespan [84]. In mammals TGFβ promotes hematopoietic stem cell quiescence by downregulating Akt activity and upregulating FoxO3 activity [85], and in glioblastoma cells TGFβ signaling induces Smad-FoxO transcriptional activation complexes that suppress proliferation [86]. In human keratinocytes, FoxO factors are required for the induction of a number of genes by TGFβ, particularly cytostatic and stress response genes [102]. Thus, it is possible that miR-25 regulate NSPCs by coordinately modulating insulin/IGF and TGFβ networks.

### Regulation of miR-106b~25 by FoxO proteins

Our experiments suggest that FoxO3 regulates miR-106b~25 in a complex manner. FoxO3 binds to a site in the first intron of miR-106b~25/Mcm7 in NSPCs. In FoxO3-null NSPCs, while Mcm7 mRNA abundance was decreased, the levels of mature miR-106b~25 members were not decreased, and were even slightly increased. Thus, FoxO3 might transcriptionally activate miR-106b~25/Mcm7, but act to repress miR-106b, miR-93, and miR-25 at a different promoter or at posttranscriptional steps like precursor cleavage, nuclear export, base editing, and degradation.

Other factors complicate our ability to define the regulation of miR-106b~25 by FoxO3. It is possible that in self-renewal culture conditions FoxO3 is bound near the promoter of miR-106b~25 but exerts control over miR-106b~25 expression only in other conditions such as differentiation, low nutrient levels, oxidative stress, or low oxygen tension. As NSPC cultures are heterogeneous, containing mixtures of stem cells, progenitor cells, and even some differentiated progeny [103,104], FoxO3 might also alter miR-106b~25 expression differently in different cell types. Such differential regulation would be consistent with FoxO3 and miR-106b~25 both promoting neuronal differentiation but having opposite effects on NSPC proliferation [13,14]. In these scenarios, FoxO3 would serve as one component of a “coincidence detector” regulating miR-106b~25, which in turn might indirectly influence FoxO activity.

## CONCLUSION

This study shows that miR-106b~25 members modulate NSPC proliferation and differentiation and could potentially be regulated by the pro-longevity transcription factor FoxO3 under some circumstances. These results suggest a role for miR-106b~25 in normal adult stem cell function, in addition to a known role in cancer cells. Understanding how miR-106b~25 and FoxO3 function in NSPCs could reveal new strategies for preventing the loss of neurogenesis in adults, particularly during aging.

## METHODS

### Constructs

For miRNA overexpression, the 725-bp segment of the mouse Mcm7 gene containing the miR-106b, miR-93, and miR-25 precursors was cloned between the XhoI and PmeI sites of the MDH1-PGK-GFP 2.0 vector [105] using the primers F: 5' -AAACTCGAGCCTGCTGGCCATTCTCCGACTTTC C-3' and R: 5' -AAAGTTTAAACGGATCTTTCTTTGCTCCAGCTTCAAGC-3'. The 350-bp segment of the mouse Mcm7 gene containing the miR-25 precursor only was cloned between the XhoI and EcoRI sites of the MDH1-PGK-GFP 2.0 vector using the primers F: 5' -AAACTCGAGCCCAGGACACAACCTCTGAT-3' and R: 5' -AAAGAATTCGAGGGGAATGAAGTCAAGGA-3'.

For luciferase assays, the 500-bp region of the mouse Mcm7 intron containing the FoxO3 binding site was cloned between the KpnI and XhoI sites of the pGL3-SV40 vector (Promega) using the primers F: 5' -AAAGGTACCGCAGTGTTCCTTTTCACAAGTCCG-3' and R: 5' -AAACTCGAGCGTGTGTAAACAGTGTCCTTCCGC-3'. Mutations in the FoxO binding sequence were made using the primers F: 5' -CCGCTCTTAATAGACAAAGAAGCACATGGGCCCAGATTCC-3' and R: 5' -GGAATCTGGGCCCATGTGCTTCTTTGTCTATTAAGAGCGG-3', and this mutated enhancer was subcloned into a new pGL3-SV40 backbone. The positive control plasmid, pGL3-SV40 containing three repeats of the FoxO3 binding site in the FasL promoter, and the FoxO3 expression plasmids were described previously [55].

### Antibodies

For immunocytochemistry, the primary antibodies used were rat anti-BrdU (AbD Serotec; 1:500), goat anti-GFP (Rockland; 1:500), rabbit anti-Tuj1 (Covance; 1:1000), rat anti-GFAP (Calbiochem; 1:1000), and mouse anti-O4 (a gift from Ben Barres; 1:1000). Fluorescent secondary antibodies were from Jackson ImmunoResearch and Molecular Probes (Invitrogen) and were used at 1:400 dilutions. The antibodies for ChIP were rabbit anti-FoxO3 “NFL” (Brunet laboratory) and rabbit IgG (Zymed).

### NSPC isolation and culture

Each NSPC culture was generated from four to eight FVB/N mice (1:1 male-female ratio). Whole brain was extracted from each animal, and the olfactory bulbs, cerebellum, and brainstem were discarded. To dissociate the forebrain tissue, brains were diced, treated at 37°C for 30 min with HBSS (Invitrogen) containing 2.5 U/ml Papain (Worthington), 1 U/ml Dispase II (Roche), 250 U/ml DNase I (Sigma), and 1X penicillin-streptomycin-L-glutamine (PSQ; Invitrogen), and then mechanically dissociated in DMEM/F12 (Invitrogen) containing 10% fetal bovine serum (FBS; Invitrogen) and 1X PSQ. NSPCs were purified from myelin with a 22.5% Percoll gradient (GE Healthcare) and then from red blood cells with a 58.5% Percoll gradient. Freshly isolated NSPCs were considered “passage 1.”

NSPCs were grown at 5% CO_2_ in a 37°C incubator at 50,000 cells/ml in Neurobasal A Medium (NBA; Invitrogen) supplemented with 1X PSQ, 1X B-27 Supplement Minus Vitamin A (B27; Invitrogen), 20 ng/ml recombinant human bFGF (PeproTech), and 20 ng/ml recombinant human EGF (PeproTech). Cells were fed every 2 days by replacing half the media and replenishing bFGF and EGF; cells were transferred to a new plate every 4 days. NSPCs grew to full neurosphere colonies every 5-8 days, and were passaged using Accutase (Millipore) for dissociation.

### miRNA overexpression by retroviral infection

HEK 293T cells were co-transfected with the expression vector MDH1-PGK-GFP 2.0 containing either miR-106b~25 or no insert (empty control) and the pCL-Eco viral packaging vector in a 2:1 ratio, using the calcium phosphate transfection method. The media was changed to NBA containing 1X PSQ and 1X B27 6-8 h later. The next day, NSPCs were dissociated and plated at 50,000 cells/ml on plates coated with 50 μg/ml poly-D-lysine (Sigma). The following day, NSPCs were infected by replacing half the media with 0.45 μm-filtered virus-containing supernatant from the 293T cultures and replenishing the growth factors. Sixteen hours later, the infection was stopped by replacing all the media with NSPC-conditioned media and fresh media in a 1:1 ratio and replenishing growth factors. NSPCs were fed every other day until they were 80% confluent, and then detached with Accutase and grown in suspension. After NSPCs had grown to full neurospheres, RNA and protein were collected, and cells were plated for proliferation assays.

### miRNA knockdown

NSPCs were plated at 100,000 cells/ml in 0.5 ml NBA containing 1X L-glutamine (Invitrogen) and 1X B27 with growth factors in a poly-D-lysine-coated well of a 24-well plate. The next day, 45 nM locked nucleic acid (LNA) oligonucleotide (Exiqon) was diluted with 100 μl Opti-MEM (Invitrogen), incubated with 1 μl Lipofectamine PLUS reagent (Invitrogen) per 1 μg nucleic acid for 5 min, and then incubated with 6 μl Lipofectamine LTX reagent (Invitrogen) per 1 μg nucleic acid for 30 min before being added to cells. The media was changed to 1 ml NBA containing 1X PSQ and 1X B27 with growth factors 4-6 h later.

### Proliferation assays

One week after retroviral infection (when NSPCs had grown to full neurospheres), NSPCs were dissociated and plated on nitric acid-treated glass coverslips (Bellco) coated with poly-D-lysine. Two days later, BrdU (EMD Biosciences) was added to a final concentration of 10 μM, or EdU (Invitrogen) was added to a 5 μM final concentration. One hour later, NSPCs were fixed in 4% paraformaldehyde in PBS for 12 min. The coverslips were blocked for 1 h with 10% donkey serum and 0.1% Triton in PBS and then incubated with goat anti-GFP antibody for 2 h. The coverslips were then refixed with 4% paraformaldehyde for 10 min and incubated with 0.4% Triton for 30 min. DNA was denatured with 2 N HCl for 10 min. After 1 h of blocking, coverslips were incubated with rat anti-BrdU antibody for 2 h. The coverslips were incubated with Texas Red donkey anti-rat and FITC donkey anti-goat secondary antibodies for 1 h. The coverslips were mounted on slides using Vectashield with DAPI (Vector Labs).

Two days after transfection with LNA probes, EdU was added to a final concentration of 5 μM. One hour later, NSPCs were fixed in 4% paraformaldehyde and 2% sucrose for 12 min. Cells were permeabilized with 0.4% Triton in PBS for 30 min and blocked with two 3% BSA (USB) rinses. Cells were then incubated in 1X Click-iT Reaction Buffer, 4 mM CuSO_4_, 1:400 Alexa Fluor 594 azide, and 200 nM Click-iT EdU Buffer Additive (Invitrogen) for 30 min. Cells were then washed with 3% BSA, rinsed with PBS, and mounted on slides using Vectashield with DAPI.

Coverslips were examined using a Zeiss Axioskop 2 Plus microscope and digital camera with AxioVision 4 software. For quantification, 3-6 random fields (about 1000-2000 cells) were counted in a blinded manner, using Metamorph 7.0 software.

### Differentiation assays

NSPCs were dissociated and plated on nitric acid-treated coverslips coated with poly-D-lysine at a density of 25,000 cells/ml. NSPCs were infected the next day, and the infection was stopped after 16 h. Two days later, NSPCs were differentiated by changing the media to NBA containing 1X PSQ, 1X B27, and 1% FBS. The media was replaced every other day. After 7 days of these differentiation conditions, NSPCs were stained for GFAP, Tuj1, or O4. For GFAP and Tuj1 staining, NSPCs were fixed in 4% paraformaldehyde and 2% sucrose. The coverslips were blocked for 1 h with 10% donkey serum and 0.1% Triton in PBS, and then incubated with rabbit anti-Tuj1 antibody for 2 h. After rinsing with PBS containing 0.01% Tween and blocking for another 15 min, coverslips were incubated with Texas Red donkey anti-rabbit or anti-rat secondary antibody for 1 h. For O4 staining, NSPCs were blocked with 5% goat serum and 7.5% BSA in PBS for 1 h and then incubated with mouse anti-O4 antibody (in 10% goat serum, 1% BSA, and 100 mM L-lysine in PBS) for 2 h. After rinsing with PBS, cells were fixed in 4% paraformaldehyde and 2% sucrose, blocked for another 15 min, and then incubated with Alexa Fluor 546 goat anti-mouse secondary antibody for 1 h. Coverslips were mounted on slides using Vectashield with DAPI. The total number of neurons on each coverslip was counted in a blinded manner, and the total number of nuclei was estimated by counting 5 random fields (about 300-600 cells) in a blinded manner.

### Target prediction

TargetScan (www.targetscan.org, version 5.1) was used to predict all conserved targets for mouse miR-25. This target list was analyzed using PANTHER (www.pantherdb.org, version 7) to compare Biological Process associations for genes in this list and the reference list, “NCBI: *M. musculus* genes,” or analyzed with GSEA Molecular Signatures Database (www.broadinstitute.org/gsea/msigdb/, version 3.0) to compute overlaps for genes in this list and “CP” (Canonical Pathways) and “C5” (GO Gene Sets). The DIANA-miRPath program (http://diana.cslab.ece.ntua.gr/pathways/) using DIANA-microT-3.0-Strict was used to predict and analyze conserved targets for mouse miR-25 in the KEGG database.

### RT-qPCR

Total RNA was extracted from NSPCs using the miRVana kit (Ambion). RNA was treated to remove genomic DNA in a reaction containing 100 ng/μl RNA, 1 U/μl RNase OUT (Invitrogen), and 10 U/μl DNase I (Invitrogen) at 37°C for 15 min and 75°C for 15 min.

miRNA expression was quantified using the miRCURY LNA miRNA PCR system or the miRCURY LNA Universal RT miRNA PCR system, according to the manufacturer's instructions (Exiqon). Samples were run in triplicate on a C1000 Thermal Cycler with the CFX96 Real-Time software (Bio-Rad), and miRNA expression was normalized to 5S RNA expression.

To quantify Mcm7 mRNA expression, RT was carried out using the High Capacity cDNA Reverse Transcription kit (Applied Biosystems). Each reaction contained 1X RT Buffer, 4 mM each dNTP, 1X Random Hexamers, 1 U/μl RNase OUT, 2.5 U/μl MultiScribe Reverse Transcriptase, and 45-90 ng/μl RNA. RT was performed at 25°C for 10 min, 37°C for 2 h, and 85°C for 5 min. Each 20-μl qPCR reaction contained 0.25 μM forward (F) Primer, 0.25 μM reverse (R) Primer, 10 μl iQ SYBR Green Supermix (Bio-Rad), and 0.625 μl RT reaction. The program used was 95°C for 10 min; 40 cycles of 95°C for 20 sec, 55°C for 20 sec, and 72°C for 45 sec. Samples were run in triplicate, and Mcm7 expression was normalized to β-actin expression. The Mcm7 primers were F: 5' -TGAACACCGGCTGATGATGG-3' and R: 5' -GGCCTCGGAAATACAACTCAA-3'. The β-actin primers were F: 5' -TGTTACCAACTGGGACGACA-3' and R: 5' -CTCTCAGCTGTGGTGGTGAA-3'.

### Chromatin immunoprecipitation

ChIP was performed as described [14] using IgG or FoxO3 antibodies. Immunoprecipitated chromatin was analyzed with qPCR: each 20-μl reaction contained 2.5 μl DNA, 10 μl iQ SYBR Green Supermix, 0.25 μM F primer, and 0.25 μM R primer. Triplicate reactions were run with the following program: 94°C for 3 min; 40 cycles of 95°C for 20 sec, 57°C for 30 sec, and 72°C for 30 sec. The primers to amplify the region surrounding the FHRE FoxO3 binding site in the Mcm7 first intron were F: 5' -TAGGCCTCCTCTGCACTCAT-3' and R: 5' -AGGAATCCTGGGCTGTGAG-3'. The negative control primers to amplify an intergenic region lacking a Forkhead binding sequence were F: 5' -GGGGGATAATGATTGCAAAA-3' and R: 5' -GCGTGGACAGAGATCTAGGC-3'. For each chromatin sample, a standard curve using five 5-fold dilutions of input chromatin was used to quantify binding at each target site in the ChIPs: linear regression (y=-ax+b) was performed on Ct versus log_5_(input), and the amount of a site in the FoxO3 ChIP relative to the IgG ChIP was calculated as 5^-ΔCt/a^, with ΔCt=Ct_FoxO3_-Ct_IgG_.

### Electrophoretic mobility shift assay

Complementary oligonucleotides (20 μM) were annealed in 100 mM NaCl by heating at 80°C for 5 min and then cooling slowly to room temperature. Annealed probe (1 μM) was labeled with 20 μCi/μl ^32^P-γ ATP and 1 U/μl T4 PNK at 37°C for 1 h. Annealed probes were purified on 15% polyacrylamide and resuspended in 1X TE pH 8.

Each binding reaction was performed in Binding Buffer (200 mM Tris-HCl pH 7.5, 200 mM KCl, 200 mM MgCl_2_, 2% NP-40, 10% glycerol, 5 mM DTT, and 500 ng/μl salmon sperm DNA) and contained 50 ng/μl GST or human FoxO3-GST, 1000 cpm/μl hot probe (5 nM FHRE probe; 3 nM positive control probe), and 0, 5, 50, or 500X competing cold probe. The reactions were incubated at room temperature for 20 min and then resolved on 4% non-denaturing PAGE at 4°C. The gels were dried and then autoradiographed for 4 days. The positive control oligonucleotides for a site bound by FoxO3 near its own promoter [106] were F: 5' -AAATAACACACACGTGTGCTGGTAAACAAGCGCGCCAGCC-3' and R: 5' -GGCTGGCGCGCTTGTTTACCAGCACACGTGTGTGTTATTT-3'. The oligonucleotides for the FHRE site within the Mcm7 intron region bound by FoxO3 in ChIP experiments were F: 5' -GGCCCATGTGCTTCTTTGTTTACTAAGAGCGGAAGCAG-3' and R: 5' -CTGCTTCCGCTCTTAGTAAACAAAGAAGCACATGGGCC-3'. The oligonucleotides for this FHRE site containing mutations in the FoxO consensus binding sequence were F: 5' - GGCCCATGTGCTTCTGTGTCTATTAAGAGCGGAAGCAG-3' and R: 5' -CTGCTTCCGCTCTTAATAGACACAGAAGCACATGGGCC-3'.

### Luciferase assays

HEK 293T cells were plated in 24-well plates at 150,000 cells/ml. The next day, they were transfected using the calcium phosphate method with 400 ng each of FoxO3 expression plasmid, pGL3-SV40 firefly luciferase plasmid, and pRL-null Renilla luciferase plasmid. Two days after transfection, cells were lysed with 0.5 ml Passive Lysis Buffer (Promega) and luciferase activity was measured with the Dual Luciferase Reporter Assay system. Triplicate transfections were averaged within each experiment, and firefly luciferase activity was normalized to Renilla luciferase activity.

### Statistical analysis

Gene expression (RT-qPCR experiments) was analyzed using one-sample two-tailed t-tests. NSPC phenotype (proliferation and differentiation assays) was analyzed using paired two-tailed t-tests. The luciferase assay experiments were analyzed with unpaired two-tailed t-tests.
